# Tracheal Rupture After Trauma: A Successful Conservative Management

**DOI:** 10.7759/cureus.32681

**Published:** 2022-12-19

**Authors:** Núria Jorge, Liliana Costa, Sara Teixeira, André Silva-Pinto, José Paiva

**Affiliations:** 1 Intensive Care Medicine Department, Centro Hospitalar Universitário de São João, Porto, PRT; 2 Infectious Diseases Department, Centro Hospitalar Universitário de São João, Porto, PRT

**Keywords:** surgical treatment, conservative treatment, airway trauma, tracheal injuries, trachea

## Abstract

Tracheobronchial injury (TBI) is a rare life-threatening injury that can result from either penetrating or blunt trauma. Treatment may be surgical or conservative, but the evidence regarding which is the best approach is still very scarce. This case report describes the successful conservative management of a 32-year-old male with a traumatic tracheal laceration. The alarming signs and symptoms, the imaging modalities of choice, the rationale behind the treatment strategy, and the most common complications are detailed here. Through this case, the authors wish to highlight the features that should lead to the suspicion of this potentially fatal traumatic injury, as well as raise awareness on how to adequately manage these patients.

## Introduction

Tracheobronchial injury (TBI) is a potentially life-threatening injury that can result from either penetrating or blunt trauma [1]. Its true incidence is unknown, as a great proportion of patients do not reach the hospital alive. Based on autopsy reports, it is estimated that 2-3% of patients who die as a result of trauma may have associated TBI [2]. The treatment approach may be surgical or conservative, depending on the patient's initial clinical presentation [3]. The authors present a case of successful conservative management of a tracheal rupture after trauma.

## Case presentation

A 32-year-old male was brought to the Emergency Department after a car accident, in which he was the driver in a side collision with a motorcycle. On admission, the airway was patent, respiratory and hemodynamic parameters were normal and the Glasgow Coma Scale was 15. Left cervical and thoracic subcutaneous emphysema and a left cervical penetrating injury with air leak stood out in the physical examination.

**Figure 1:**
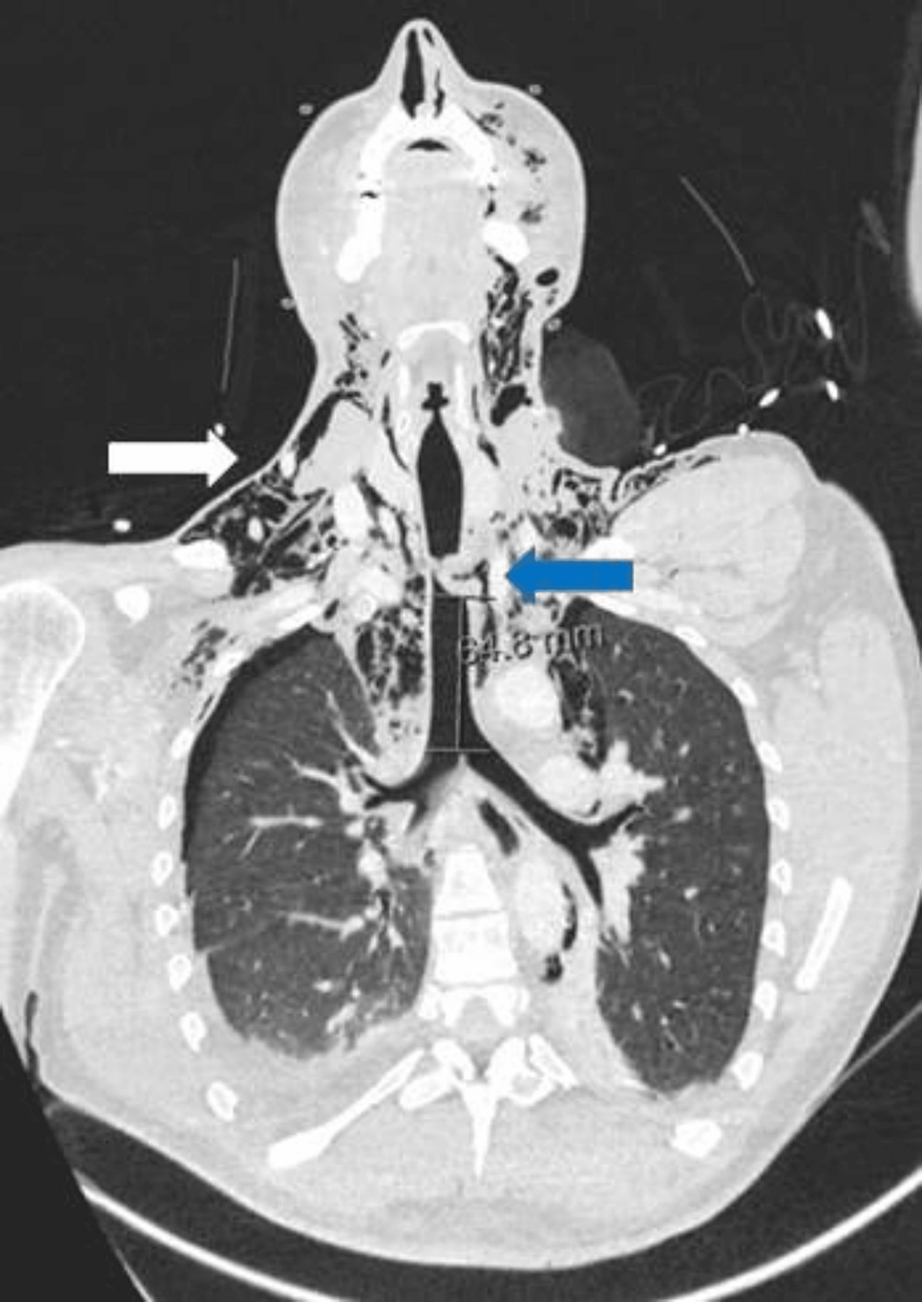
Admission thoracic CT: Left anterolateral tracheal laceration in the thoracic portion (blue arrow); subcutaneous emphysema (white arrow)

**Figure 2:**
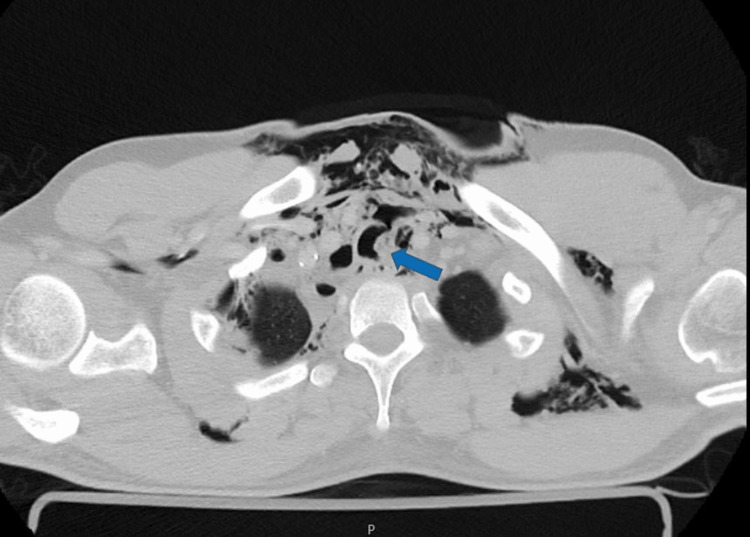
Admission thoracic CT: Tracheal laceration (axial view)

**Figure 3:**
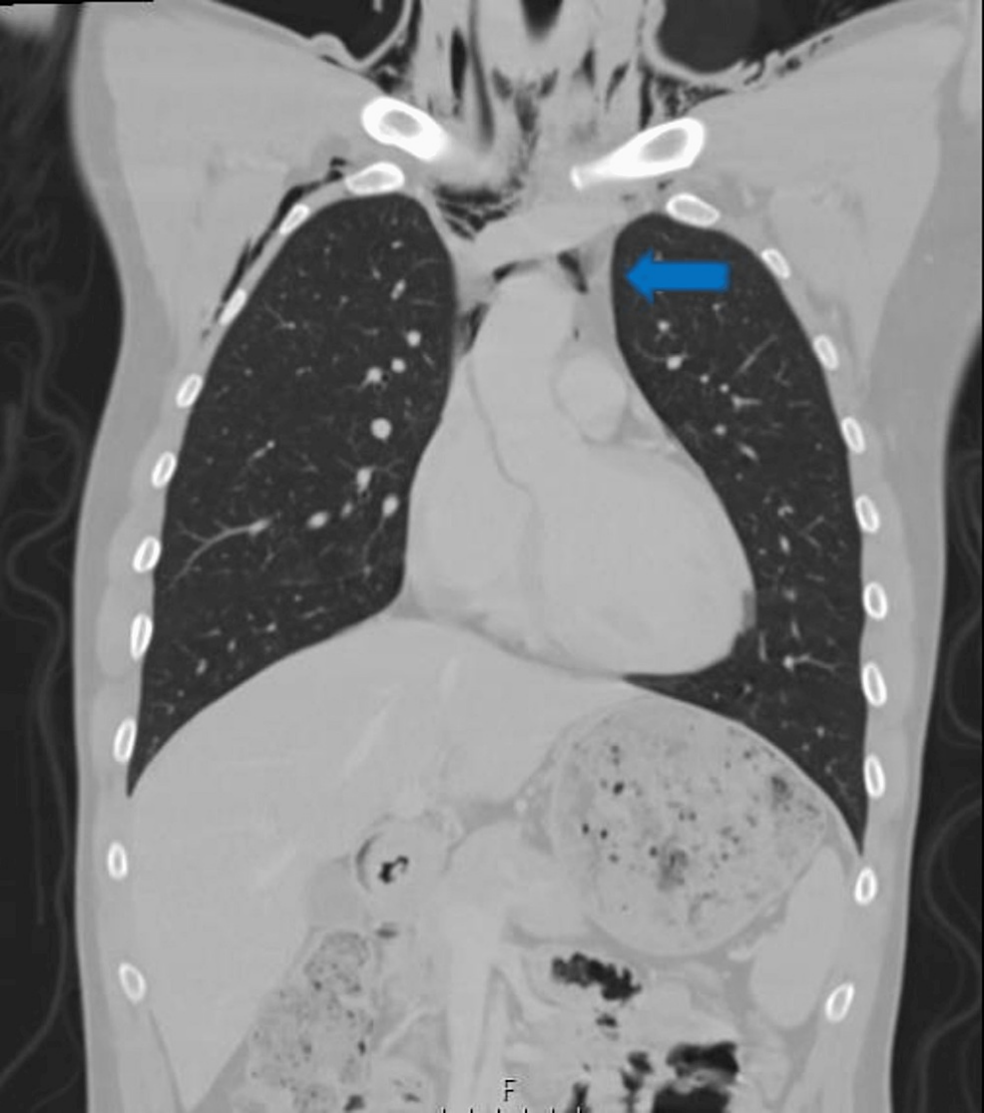
Admission thoracic CT: Pneumomediastinum

Cerebral and cervical computed tomography (CT) were normal, except for the subcutaneous emphysema in latero-cervical and pre-vertebral tissues. Facial CT flagged an aligned fracture of the left ramus of the mandible. Thoracic CT (Figures 1-3) revealed extensive thoracic subcutaneous emphysema, pneumomediastinum, lung contusions in the lower lobes, and a left anterolateral tracheal laceration in the thoracic portion. 

A multidisciplinary team comprising Otolaryngology, Cardiothoracic Surgery, and Intensive Care Medicine was promptly assembled to evaluate the clinical scenario. Since there was no respiratory distress or hemodynamic instability, a decision to follow a conservative approach was made, and the patient was admitted to an Intensive Care Unit (ICU) for close monitoring.

**Figure 4:**
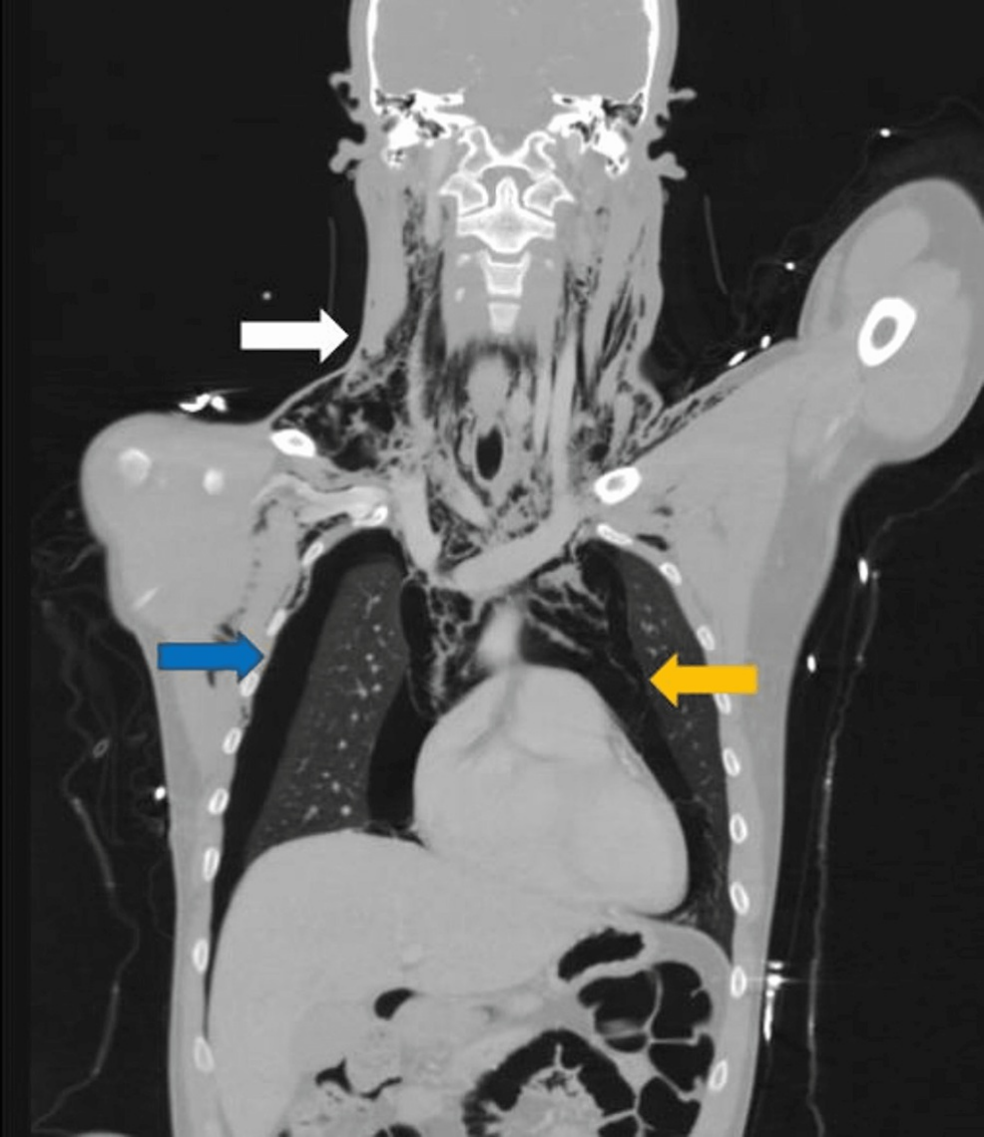
First ICU day thoracic CT: Bilateral pneumothorax, more pronounced in the right lung field (blue arrow), aggravated subcutaneous emphysema (white arrow), and pneumomediastinum (yellow arrow)

**Figure 5:**
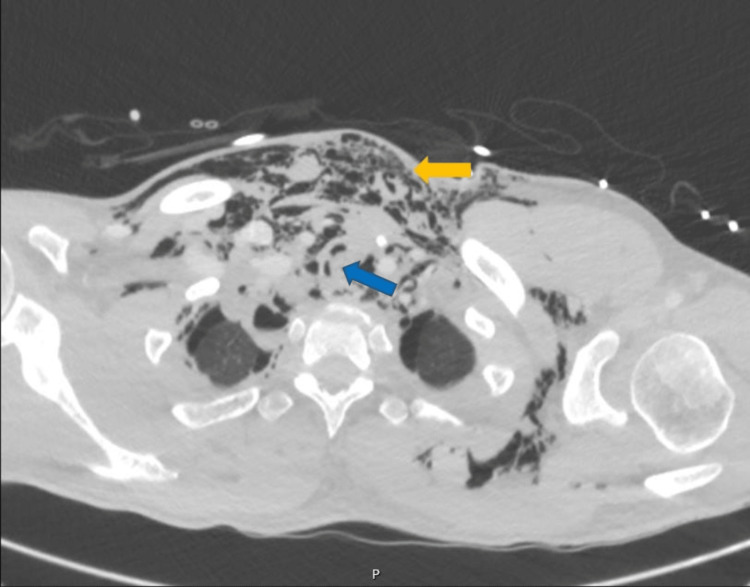
First ICU day thoracic CT: Marked reduction of the tracheal lumen, less than 3 mm (blue arrow), more expressive subcutaneous emphysema (yellow arrow)

On the following day, the patient was started on nasal oxygen to maintain the ratio of arterial oxygen partial pressure to fractional inspired oxygen (PaO2/FiO2) above 300 mmHg, without respiratory distress. Another thoracic CT was performed and revealed bilateral pneumothorax, aggravated subcutaneous emphysema, and pneumomediastinum (Figure 4). There was also an important change in the morphology of the trachea with significant bulging of the left lateral wall, leading to a marked reduction of the tracheal lumen at this level (less than 3 mm) (Figure 5).

A chest tube was placed, and a control chest X-ray confirmed the expansion of the right lung. Multimodal analgesia with intravenous paracetamol (1 g every 6 hours), tramadol perfusion (300 mg/day), and ketorolac (30 mg every 8 hours), was necessary to control cervical and thoracic pain.

Close monitoring of clinical and laboratory signs of respiratory dysfunction was preconized. Upon reassessment, Pneumology reiterated that there was no indication for bronchoscopy, due to the potential risk of adversely affecting the lesion and the clinical stability of the patient, but in case of clinical deterioration and need for orotracheal intubation, that should be performed through this technique. The extracorporeal membrane oxygenation (ECMO) team was signalled as an alternative management route if an unfavourable evolution occurred.

On the fifth ICU day, the patient presented a productive cough, fever, and worsening PaO2/FiO2 ratio. Amoxicillin/clavulanic acid (1,2 g every 8 hours) was initiated for presumed infection of pulmonary contusions. Oxygen therapy was maintained by nasal cannula, with a maximum fraction of inspired oxygen (FiO2) of 36%, without the need for ventilatory support. 

**Figure 6:**
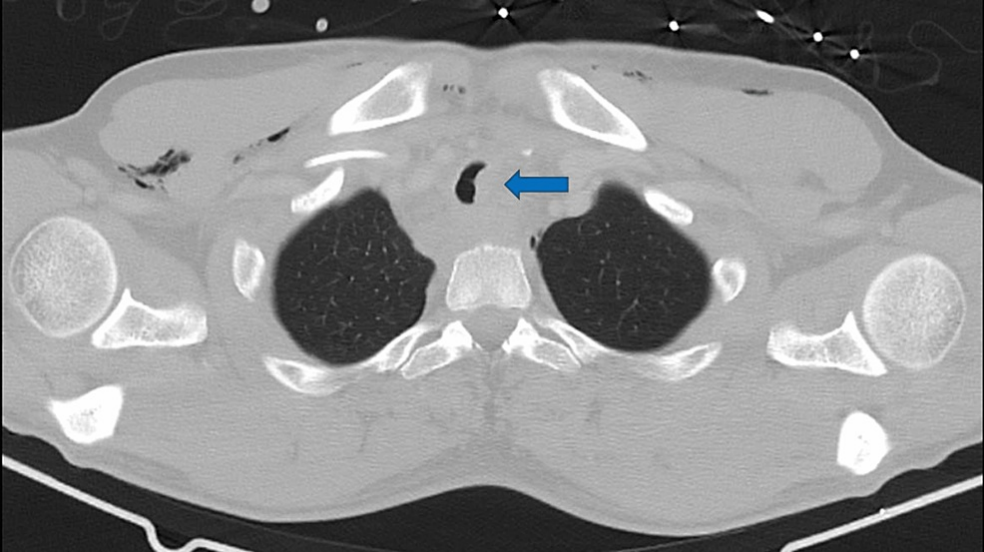
Discharge thoracic CT: Remaining bulging of the left lateral tracheal wall

**Figure 7:**
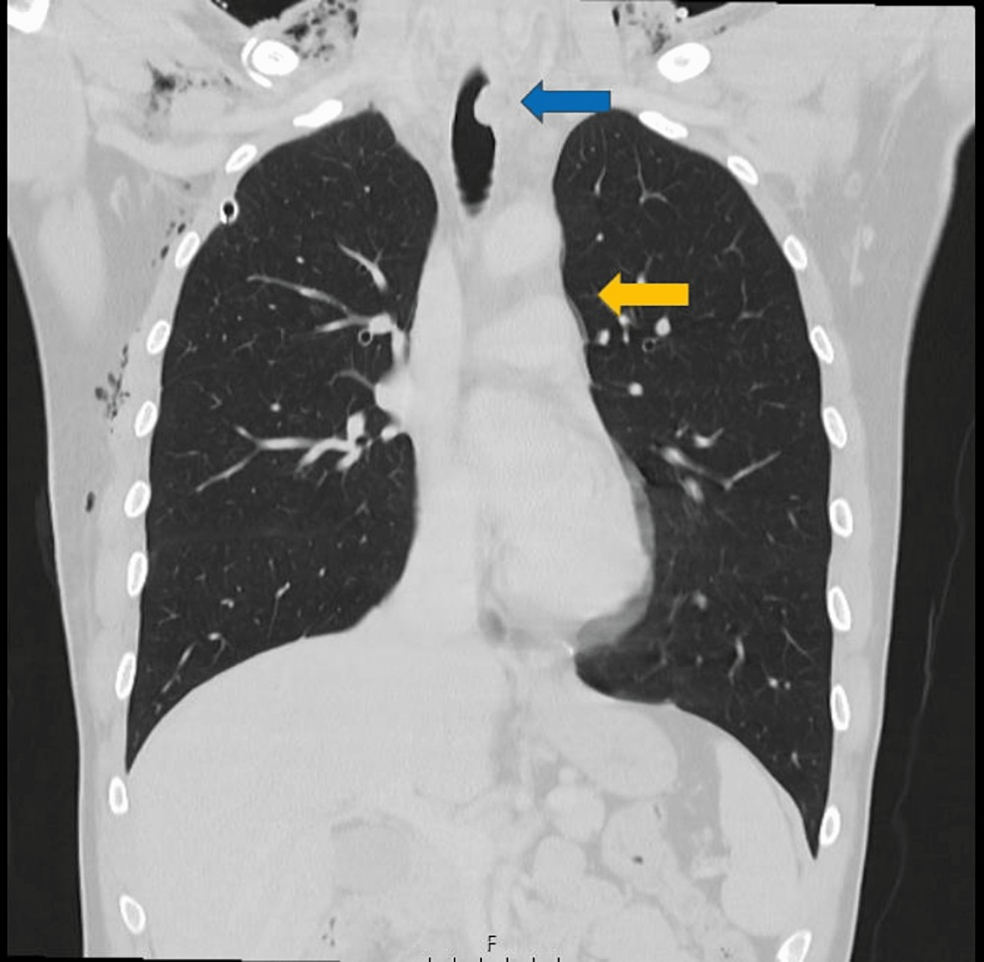
Discharge thoracic CT: Remaining bulging of the left lateral tracheal wall (blue arrow), subcutaneous emphysema and pneumomediastinum reduction (yellow arrow)

During the ICU stay, he presented clinical and imagiological stability and was constantly reassessed by Cardiothoracic Surgery and Pneumology. Oxygen therapy was titrated until suspension on day 6, and the antibiotic was maintained for 8 days. A thoracic CT was done on the 13th day (Figures 6-7) and showed remaining bulging of the left lateral wall of the proximal trachea causing a minimum useful airway lumen of 7 mm, and small volume pneumomediastinum. Mild thoracic subcutaneous emphysema persisted. 

The chest tube was removed on the 13th day, uneventfully.

On the 14th day, after a multidisciplinary discussion, he was discharged from the ICU. He stayed hospitalized for a further five days in the Maxillofacial Surgery ward due to the mandibular fracture. Due to the risk of submitting the patient to a surgical procedure and the aligned nature of the mandible's fracture, it was decided to follow a conservative approach for this problem. 

The patient was discharged home with an indication for a liquid diet and reassessment in two weeks. A difficult airway card was handed out prior to discharge.

The follow-up period was uneventful. Re-examination by Cardiothoracic Surgery, after two months, included a bronchoscopy evaluation, which revealed tracheal stenosis in the medial third of the left lateral wall, causing a 30% decrease in calibre. 

Maxillofacial Surgery maintained the decision not to surgically intervene and physical rehabilitation was started. Three months later, he still maintained rehabilitation and follow-up by Maxillofacial Surgery.

## Discussion

Tracheobronchial injury may be caused by penetrating or blunt trauma to the neck and chest. The incidence is still unknown, but if victims who die at the scene are considered, it is estimated to occur in 0.5% to 2% of the victims with neck and chest trauma [4]. Considering only penetrating neck injuries, the incidence of cervical tracheal injury is 3% to 6% [5]. 

Airway injuries are rare events, even in trauma centres, and the lack of experience can lead to delayed diagnosis [6]. Prompt diagnosis is mandatory to avoid early fatal outcomes. Dyspnoea and respiratory distress are frequent symptoms (59-100%) [4] and the most common signs include subcutaneous emphysema (35-85%) and pneumothorax (20-50%) [5]. Air escape from a penetrating neck wound is pathognomonic of airway laceration, occurring in approximately 60% of the patients with tracheal injury by penetrating trauma [5]. 

The initial diagnostic workout is not different from other trauma evaluations. However, the role of CT is not well established. CT can detect signs of airway trauma, like pneumothorax and pneumomediastinum, but it is less specific to detect airway injury. As such, a negative CT scan does not exclude the presence of airway trauma [4,5]. Bronchoscopy is the exam of choice to diagnose and characterise airway injuries [6,7]. 

The patency of the airway is the priority of the treatment pathway. Spontaneous breathing approaches and bronchoscopy-guided intubation are the preferred routes to avoid procedure complications [4]. The injury can then be managed conservatively or surgically. 

Classically, early surgical repair was recommended for all tracheal injuries. This recommendation is based on the idea of complication avoidance, like mediastinitis or tracheal stenosis [8]. However, there are no randomised trials supporting this approach over the conservative approach [8]. 

Conservative management is now well described, mainly in iatrogenic injuries and ventilated patients [4]. Requirements for following this approach are established, namely small lacerations (< 2 cm), cuff inflated distally to the injury, ventilation with positive end-expiratory pressure (PEEP) and low tidal volumes, no signs of related infection and controlled pneumothorax and subcutaneous emphysema [4]. Some studies have shown equally good outcomes with the conservative strategy in traumatic tracheal injuries when these criteria were met, but no randomised trials have validated this data [9,10].

A small lesion treated conservatively has a greater possibility of success and lesions larger than 4 cm are considered risky to approach this way [9]. Early diagnosis has a great impact on the treatment choice. The primary surgical approach in early diagnosis is defended by various surgical groups, as a late diagnosis is associated with the worst tissue conditions and possible worst outcomes [6,9]. However, if the previously described criteria are met, the surgical approach can be delayed and the indication for surgery can be established in the immediate follow-up of a lesion initially treated conservatively [9]. Tracheal intubation, guided by bronchoscopy, can be the treatment choice to bridge tracheal lacerations, not only in proximal localizations. In these cases, serial bronchoscopies should be performed to confirm tube position, to perform bronchial toilette, to follow lesion evolution, and to monitor the need for surgical repair [8,10]. 

The objectives of the surgical approach include repair of the airway defect, prevention of mediastinal involvement, and avoidance of healing complications [4]. The procedure technique depends on the lesion site. Comorbidities and underlying conditions, like multiple traumas, are factors to consider in the morbimortality of these patients [10].

Mortality after surgical repair is described as high as 42% [7]. In fact, data analyses of patients with post-intubation tracheal lesions revealed that a high mortality rate may result from underlying clinical conditions but also from the method of treatment, especially in surgically managed patients [10]. 

In this clinical case, we favoured a conservative approach. A vision that includes clinical rather than morphological features for surgical intervention is therefore not an invalid option in selected patients. Strict monitoring of respiratory failure and infection development should be undertaken [4], justifying the hospitalisation of these patients in intensive care units. The surgical approach can always be an option after initial conservative treatment [9].

The same rationale was followed regarding the therapeutic strategy for respiratory insufficiency. Mechanical ventilator support was avoided to evict related complications, such as elevated airway pressure or infections. Spontaneous breathing was tolerated once the patient remained clinically stable.

Lastly, late sequelae such as airway stenosis and recurrent pulmonary infections should be accessed. After airway injury, granulation tissue and stenosis develop within 1 to 4 weeks, leading to chronic complications [5]. Bronchoscopic assessment is important to diagnose this late involvement, and follow-up consults after discharge should be undertaken. 

## Conclusions

Tracheobronchial trauma is a rare and often fatal entity. The treatment approach is still controversial since there are no randomised studies comparing the two possible paradigms: surgical or conservative.

Surgical repair as the primary approach is not always necessary and conservative management can be a viable choice. Clinical monitoring is of paramount importance, as the development of airway compromise, ventilatory failure, or signs of infection, dictates the need for surgical intervention. Importantly, the cooperation of a multidisciplinary team throughout the clinical course is crucial for a favourable outcome.
